# Glucocorticoid-Treated Mice Are an Inappropriate Positive Control for Long-Term Preclinical Studies in the *mdx* Mouse

**DOI:** 10.1371/journal.pone.0034204

**Published:** 2012-04-03

**Authors:** Arpana Sali, Alfredo D. Guerron, Heather Gordish-Dressman, Christopher F. Spurney, Micaela Iantorno, Eric P. Hoffman, Kanneboyina Nagaraju

**Affiliations:** 1 Research Center for Genetic Medicine, Children’s National Medical Center, Washington DC, United States of America; 2 Department of Integrative Systems Biology, George Washington University School of Medicine and Health Sciences, Washington DC, United States of America; 3 Division of Cardiology, Children’s National Medical Center, Washington DC, United States of America; University of Edinburgh, United Kingdom

## Abstract

**Background:**

*Dmd^mdx^* (*mdx*) mice are used as a genetic and biochemical model of dystrophin deficiency. The long-term consequences of glucocorticoid (GC) treatment on dystrophin-deficient skeletal and heart muscle are not yet known. Here we used systematic phenotyping to assess the long-term consequences of GC treatment in *mdx* mice. Our investigation addressed not only the effects of GC on the disease phenotype but also the question of whether GCs can be used as a positive control for preclinical drug evaluations.

**Methods and Findings:**

We performed nine pre-clinical efficacy trials (treated N = 129, untreated N = 106) of different durations in 9-to-50-week-old dystrophic *mdx* mice over a 3-year time period using standardized methods. In all these trials, we used either 1 mg/kg body weight of prednisone or 5 mg/kg body weight of prednisolone as positive controls to compare the efficacy of various test drugs. Data from untreated controls and GC-treated mice in the various trials have been pooled and analyzed to assess the effects of GCs on dystrophin-deficient skeletal and cardiac muscles of *mdx* mice. Our results indicate that continuous GC treatment results in early (e.g., at 50 days) improvements in normalized parameters such as grip strength, motor coordination and maximal *in vitro* force contractions on isolated EDL muscle, but these initial benefits are followed by a progressive loss of muscle strength after 100 days. We also found a significant increase in heart fibrosis that is reflected in a significant deterioration in cardiac systolic function after 100 days of treatment.

**Conclusion:**

Continuous administration of prednisone to *mdx* mice initially improves skeletal muscle strength, but further therapy result in deterioration of muscle strength and cardiac function associated with enhanced cardiac fibrosis. These results suggest that GCs may not serve as an appropriate positive control for long-term *mdx* mouse preclinical trials.

## Introduction

Glucocorticoids (GCs) are among the most widely prescribed drugs because of their anti-inflammatory and immunosuppressant properties. Randomized controlled studies have indicated that GC therapy in Duchenne muscular dystrophy (DMD) improves muscle strength, ambulation, and respiratory function and decreases scoliosis in short-term studies [1], [2]. GC treatment elicited significant improvements in whole-body strength as well as measurable incremental increases in running endurance in *mdx* mice. This treatment also appeared to protect *mdx* mice from the stressful effects of continuous running, as determined by strength and muscle fiber diameter [3].

However, the use of GCs in DMD remains controversial, in part because of their significant side effects, including osteoporosis, growth retardation, and immune suppression. Furthermore, the beneficial effects of GCs may depend on pathways other than those associated with their well-documented anti-inflammatory properties. Studies of other immunosuppressive drugs, such as azathioprine, have shown decreases in inflammatory infiltrates in DMD skeletal muscle similar to those produced by prednisone, but these drugs did not show the improved muscle strength associated with prednisone [4]. Golumbek et al. have also demonstrated that *mdx* mice deficient in mature T and B lymphocytes do not show any functional improvements in disease phenotype [5]. These studies suggest that some of the therapeutic effects of GCs are independent of their immunosuppressive properties. It is currently unclear when the beneficial effects of GCs wane and further therapy leads to adverse effects in dystrophin deficiency.

The purpose of the current study was to a) comprehensively analyze the effects of chronic continuous GC administration on dystrophin-deficient *mdx* mice and b) evaluate whether GCs can be used as positive controls in preclinical drug efficacy trials in the *mdx* mouse model. In this study, we used a wide variety of behavioral, functional, histological, biochemical, imaging, and molecular assays to comprehensively assess the effects of GCs administered for 50, 100, or 180 days to the *mdx* mice. We found that treatment with GCs resulted in weight loss and an initial, partial improvement in grip strength but a subsequent progressive loss of strength, catabolic effects, and deterioration in functional, histological, and biochemical measures in dystrophin-deficient skeletal and cardiac muscle.

## Methods

### Animal Care

All mice were handled according to local Institutional Animal Care and Use Committee (IACUC) guidelines. Generally, 8- to 10-week-old female C57BL/10ScSn-Dmd*^mdx^*/J (*mdx*) mice weighing approximately 20–25 g were purchased from The Jackson Laboratory (Bar Harbor, ME). All mice were housed in an individually ventilated cage system with a 12-h light-dark cycle and received standard mouse chow (Harlan Teklad) and water *ad libitum*. All mice were allowed to rest for at least 7 days in the facility before acclimatizing them on the instruments and taking baseline readings for behavioral assays.

### Study Design

We tested the efficacy of nine drugs over a period of 3 years (July 2006 to July 2009) in the mdx (Duchenne muscular dystrophy) mouse model. In each trial, we used a prednisone arm to assess and compare the efficacy of the test drugs to that of current standard clinical therapy. In order to address the issue of the efficacy of prednisone in mdx mice, we pooled the data from the prednisone-treated groups from all nine trials (n = 129) and compared the aggregate results to those for untreated controls (n = 106) after 50, 100, and 180 days of chronic administration of prednisone. We would like to note that we have used prednisone as well as prednisolone preparations and administered the drugs by various routes (subcutaneous pellets, oral chow, and daily syrup). Five trials used subcutaneous prednisone, three used prednisolone chow, and one used prednisolone syrup. Since all of these modes of prednisone/prednisolone administration resulted in similar weight loss, we have combined them into one group and designated this aggregate as the GC-treated group.

### Drug Treatment

mdx mice were treated with GC in three different ways: 1) Prednisone (Innovative America, Inc) was administered in the form of slow-release subcutaneous pellets at 1 mg/kg/day; for studies with a 180-day treatment period, two pellets were used per mouse, with the second pellet being implanted after 3 months. 2) Prednisolone at 5 mg/kg/day in syrup form was given orally once per day. 3) Prednisolone (5mg/kg body weight) was administered in a custom-made mouse chow research diet (Harlan Teklad) for 50, 100, or 180 days. Actual diet consumption of the drug-treated group varied in 100- and 180-day treatment periods, and the average daily consumption of prednisolone was 3.77–4.15 mg/day.

### Behavioral and Functional Assessments

In order to unmask the disease phenotype, we subjected mdx mice to treadmill exercise (a 30-min run on a horizontal treadmill at 12 m/min, twice a week) as described previously [6]. Grip strength, motor coordination (Rota-Rod), and open field activity were assessed as detailed previously [6], [7], [8]. For echocardiography, mice were anesthetized with 1–2% isoflurane in 100% oxygen, and scanning was performed over 20 min using a high-frequency ultrasound probe (RMZ 702a, Vevo 770, VisualSonics, Toronto, Canada) as previously described [8]. Qualitative and quantitative measurements were made offline using analytic software (VisualSonics, Toronto, Canada). For in vitro muscle force testing, the extensor digitorum longus (EDL) muscle from the right hindlimb was removed from an anesthetized mouse and placed vertically in a bath containing buffered mammalian Ringer solution (25^0^C) and bubbled with 95% O_2_–5% CO_2_. The distal tendon of the muscle was tied securely to the lever arm of a servomotor/force transducer (model 305B, Aurora Scientific), and the proximal tendon was fixed to a stationary post in the bath. The muscle was stimulated between two stainless steel plate electrodes. At optimal muscle length, the force developed was measured during trains of stimulation (300 ms) with increasing frequencies until the highest plateau was achieved. The force generated to obtain the highest plateau was established as the maximal force generated by the muscle and expressed in milliNewtons (mN). The specific force, expressed as kN/m^2^, was obtained by dividing the maximal force by the physiologic cross-sectional area of muscle. The physiologic cross-sectional area was calculated by dividing the muscle mass by the fiber length and density (1.056 kg/m^3^) of the muscle tissue. The fiber length was established as 0.71 of the length of the EDL.

### Histology

Mice in each of the nine drug trials were sacrificed at different ages, and the skeletal muscles were collected for hematoxylin and eosin staining (H&E). The muscles were stored in formalin for H&E staining. Serum was also acquired from these mice for use in estimating creatine kinase levels. For H&E staining, the gastrocnemius muscle was stained as previously described [6]. For quantification, five non-overlapping representative fields of the stained tissue sections were imaged under a light microscope (20X), and a digital image for each field was obtained using computer software (Olympus America Inc., Center Valley, PA). The digital images were loaded into Image J (NIH) with an additional plug-in to count cells. In brief, the total fibers present, total fibers with central nuclei, regenerating fibers (basophilic fibers), degenerating fibers, and inflammation (defined as an interstitial group of 10 smaller inflammatory cells with dark-blue nuclei in one high-power field) were assessed. Quantitative procedures were performed as detailed previously [6].

### Quantification of Fibrosis

Paraffin sections were stained with Sirius Red stain (Sigma-Aldrich, St. Louis, MO) and counter-stained with hematoxylin to visualize nuclei. The tissue was imaged under a light microscope using a 4X objective, and a digital image was obtained using computer software (Olympus C.A.S.T. Stereology System, Olympus America Inc., Center Valley, PA). The digital images were processed using Image J (NIH), with an additional threshold color plug-in to process .jpeg images. The percentage of the fibrotic area corresponding to the area stained in red was compared to the total area of the tissue section, and the results were expressed as % fibrosis.

### Statistical Analysis

Multiple traits were analyzed in the *mdx* mice, comparing the levels in mice treated with GC to those in control (non-drug-treated) mice. All traits not meeting the assumption of normality were transformed using either a log (total distance, CK level, and cardiac fibrosis) or square root (EDL weight) transformation. Those traits for which transformation did not produce the desired normality (Rotarod, rest time, and vertical activity) were subsequently analyzed using alternative methods such as quantile (or median) regression models with age, trial, and weight covariates. The mean levels of each trait at baseline were compared in the treated and untreated mice to determine whether any differences in the groups might have existed at the outset of the study. Comparisons of normally distributed and transformed traits were made using ANCOVA models with age, trial, and weight covariates. Comparisons of non-normally distributed traits were made using quantile (or median) regression models with age, trial, and weight covariates. Comparisons of mean levels of each trait were also tested at 50, 100, and 180 days of prednisone treatment. The same models were used as in the baseline measures, with the addition of a covariate describing the actual number of days each mouse was on GC treatment. Untreated mice were assigned the same number of actual days on GC as their analogous treated group in order to adequately model both treatment groups.

## Results

### Effect on Body Weight

**Figure 1:**
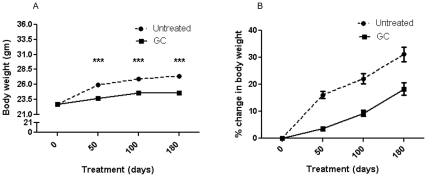
**Effect of treatments on body weight.** (**A**) *mdx* mice received no treatment (N = 84, 83, and 44) or GC treatment (N = 92, 89, and 38) for 50, 100, or 180 days, respectively, *** p = <0.001. (**B**) Comparison of the % rate change in body weight of untreated and GC-treated mice at 50, 100, and 180 days. The rate at which body weight changed was established in the first 50 days, when the change occurred in the untreated mice at a much faster rate (<0.0001). The rate remained greater than that for the treated mice, but the change over time was the same for both groups. Error bars indicate mean+/- SEM.

Body weight (BW) decreased significantly in mice that received GCs for 50, 100, or 180 days, as compared to untreated *mdx* mice; the rate of BW gain in the GC-treated mice was significantly lower than that in the untreated *mdx* mice (Figure 1A). The loss in BW gain occurred within 50 days of treatment, and the change in the rate of gain after 50 days was not significant between the treated and untreated groups (Figure 1B). There were no significant differences in the weights of the gastrocnemius and soleus muscles at 50, 100, and 180 days, but the EDL muscle weight was significantly decreased at 100 days in the GC-treated mice (p<0.002; Table 3). The spleen weights of the GC-treated mice showed a significant decrease at 100 days (p<0.05), but not at 50 or 180 days of treatment (Table 1). In contrast to the results for skeletal muscle, there was a significant and consistent increase in the heart weight of the GC-treated mice at 100 (p<0.001) and 180 (p = 0.001) days (Table 1).

**Table 1:** Weight of muscles and spleen in untreated and GC-treated mice.

Time(days)	Tissues	GC treated group		Untreated group		p-value for treatment group
		N	Mean ± SEMWeight (mg)	N	Mean ± SEMWeight (mg)	
∼50	Gastrocnemius	11	135.6±2.7	9	138.9±3.0	0.424
	Soleus	11	10.2±0.4	9	9.6±0.4	0.304
	Heart	11	106.6±4.7	9	101.6±5.2	0.486
	Spleen weight	11	82.3±3.2	9	83.9±3.5	0.737
∼100	Gastrocnemius	45	143.4±2.1	41	144.6±2.2	0.734
	Soleus	45	9.9±0.2	41	9.7±0.2	0.596
	Heart	45	120.8±2.3	41	104.8±2.4	<0.001
	Spleen	45	87.3±1.9	41	93.5±2.0	0.047
∼180	Gastrocnemius	36	130.9±2.3	37	136.5±2.2	0.199
	Soleus	36	9.2±0.2	37	8.9±0.2	0.468
	Heart	35	120.6±3.2	38	103.4±3.1	0.001
	Spleen	36	92.2±3.3	38	90.5±3.2	0.742

### Effect on Grip Strength, Motor Coordination, and Behavioral Measurements

**Figure 2:**
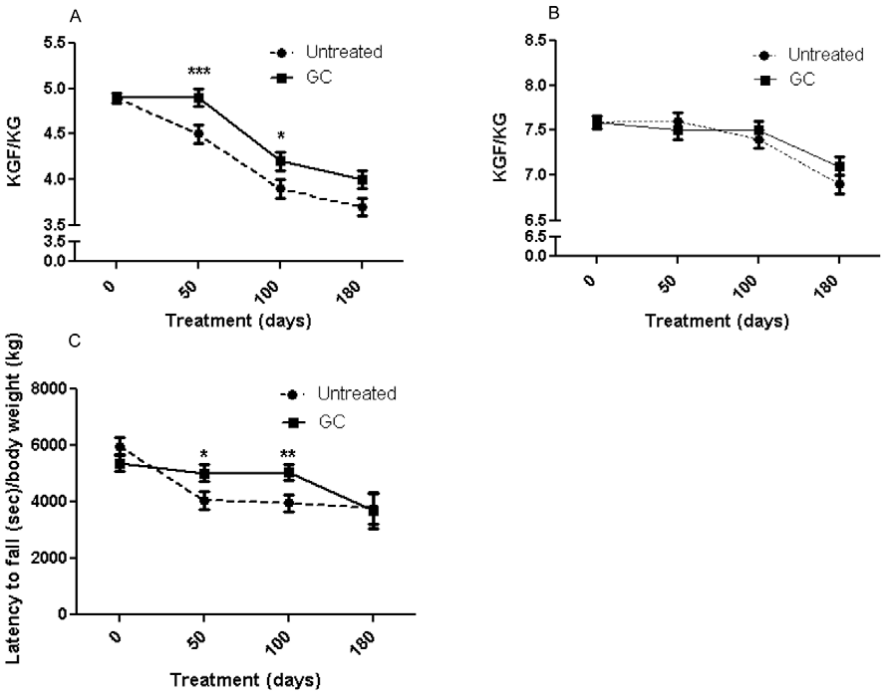
Effect of treatments on normalized grip strength. (**A**) Forelimb grip strength and (**B**) Hindlimb grip strength were measured using a grid at 50, 100, and 180 days of age. (**C**) Latency to fall normalized to body weight was measured using a rotating rod. *mdx* mice received no treatment (N = 84, 82, and 44) or GC treatment (N = 92, 87, and 38) for 50, 100, or 180 days, respectively. Error bars indicate mean +/- SEM. ** p  =  <0.01 and * p  =  <0.05.

There were no significant differences between the groups in terms of the maximal forelimb or hindlimb grip strength at 50, 100, or 180 days. Both groups showed a decline in grip strength over time (Table 2). However, the treated mice had significantly higher normalized forelimb strength than did controls at 50 (p<0.05) and 100 (p<0.05) days, but not at 180 days (Figure 2A). Normalized hindlimb grip strength did not differ significantly between the groups (Figure 2B). There was a significant increase in motor coordination performance in the treated groups at 50 (p<0.05) and 100 (p<0.01) days, but not at 180 days, in terms of the latency to-fall normalized to body weight on the Rotarod (Figure 2C). Open-field behavioral activity parameters such as horizontal activity, total distance and rest time were not significantly different between the groups, with the exception of vertical activity, which was significantly higher in the GC-treated mice at the 180-day time point (p<0.04;Table 2). However, normalization of behavioral parameters to body weight also resulted in significantly improved behavioral activity in the treated groups at 50 days and 100 days, and many of these benefits vanished by 180 days.

**Table 2:** Grip strength, motor, and behavioral measurements in untreated and GC-treated mice

Time (days)	Measurement	GC treated group		Untreated group	
		N	Mean ± SEM	N	Mean ± SEM
∼50	GSM – forelimb (KGF)	92	0.118±0.002	84	0.116±0.002
	GSM – hindlimb (KGF)	92	0.183±0.003	84	0.192±0.003
	Normalized GSM – forelimb (KGF/KG)	92	4.9±0.1	84	4.5±0.1#
	Normalized GSM – hindlimb (KGF/KG)	92	7.5±0.1	84	7.6±0.1
	Rotarod* (sec)	92	117±5	84	113±5
	Normalized Rotarod* (sec/kg)	92	5027±300	84	4064±314#
	Horizontal activity (units)	74	1357±41	72	1379±42
	Normalized horizontal activity (units/kg)	74	58243±1629	72	53048±1663#
	Total distance** (cm)	74	5.6±0.1	72	5.6±0.1
	Normalized total distance** (cm/kg)	74	9.4±0.1	72	9.3±0.1
	Rest time * (sec)	74	565.0±1.4	72	564.2±1.5
	Normalized Rest time * (sec/kg)	74	23841±200	72	21973±202#
	Vertical activity * (units)	74	33.9±3.1	72	29.7±3.2
	Normalized vertical activity * (units/kg)	74	1425±111	72	1162±116
∼100	GSM – forelimb (KGF)	87	0.102±0.002	82	0.103±0.002
	GSM – hindlimb (KGF)	87	0.185±0.003	83	0.194±0.003
	Normalized GSM – forelimb (KGF/KG)	87	4.2±0.1	82	3.9±0.1#
	Normalized GSM – hindlimb (KGF/KG)	87	7.5±0.1	83	7.4±0.1
	Rotarod* (sec)	87	102±6	82	111±6
	Normalized Rotarod* (sec/kg)	87	5060±281	82	3958±294#
	Horizontal activity (units)	87	1661±54	82	1597±56
	Normalized horizontal activity (units/kg)	87	67102±1969	82	60554±2028#
	Total distance** (cm)	87	6.0±0.1	82	6.0±0.1
	Normalized total distance** (cm/kg)	87	9.7±0.0	82	9.5±0.1#
	Rest time * (sec)	87	556.1±3.0	82	564.4±3.1
	Normalized Rest time * (sec/kg)	87	23047±368	82	20854±377#
	Vertical activity * (units)	87	38.3±5.1	82	35.1±5.4
	Normalized vertical activity * (units/kg)	87	1409±112	82	1592±116
∼180	GSM – forelimb (KGF)	38	0.097±0.002	44	0.102±0.002
	GSM – hindlimb (KGF)	38	0.180±0.003	44	0.185±0.003
	Normalized GSM – forelimb (KGF/KG)	38	4.0±0.1	44	3.7±0.1
	Normalized GSM – hindlimb (KGF/KG)	38	7.1±0.1	43	6.9±0.1
	Rotarod* (sec)	38	95±14	44	103±13
	Normalized Rotarod* (sec/kg)	38	3676±607	44	3786±559
	Horizontal activity (units)	38	1358±63	44	1311±57
	Normalized horizontal activity (units/kg)	38	51929±2062	44	50292±1916
	Total distance** (cm)	38	5.7±0.1	44	5.7±0.1
	Normalized total distance** (cm/kg)	38	9.4±0.1	44	9.3±0.1
	Rest time * (sec)	38	562.5±4.6	44	563.7±4.0
	Normalized Rest time * (sec/kg)	38	23328±369	44	19923±349#
	Vertical activity * (units)	38	28.5±2.5	44	20.8±2.2#
	Normalized vertical activity * (units/kg)	38	983±107	44	785±103

* Analyzed using quantile regression due to non-normality

** Data transformed to conform to normality (total distance, move time, CK level, and cardiac fibrosis were log transformed; EDL weight was square transformed)

#Significantly different p values for treatment groups

### Effect on Heart Function and Fibrosis

**Figure 3:**
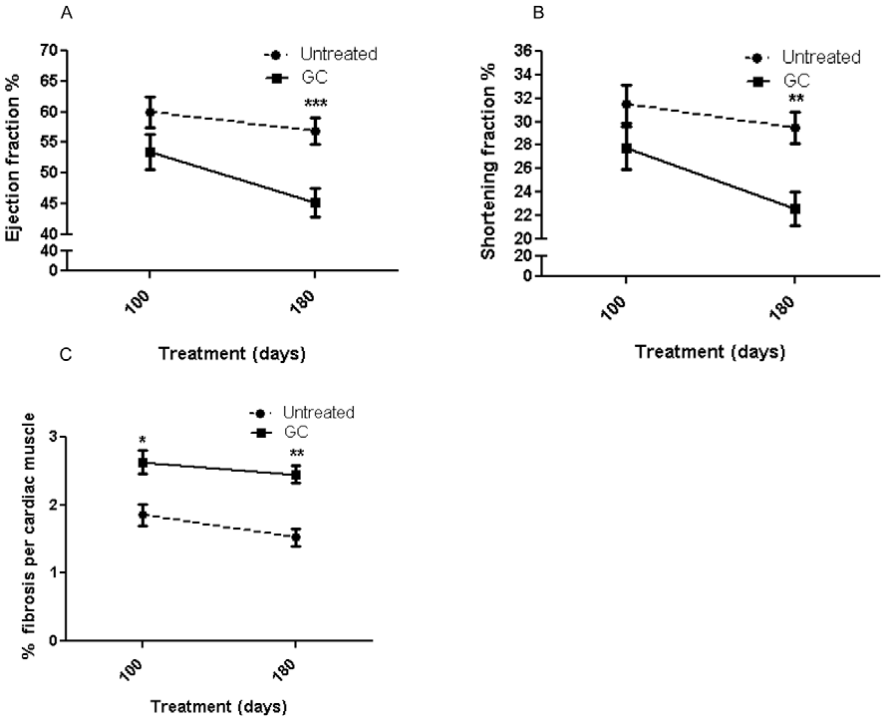
**Effect of treatments on cardiac function and fibrosis.** (**A**) Ejection fraction (%) and (**B**) shortening fraction (%) were assessed by echocardiography in mice who received no treatment (N = 30, 21) or GC treatment (N = 25, 19) for 100 or 180 days, respectively. (**C**) Sirius Red staining of formalin-fixed heart sections was used to estimate collagen content. *mdx* mice received no treatment (N = 12, 13) or GC treatment (N = 11, 14) for 100 or 180 days, respectively. Error bars indicate mean +/- SEM. **p  = <0.01 and ***p  = <0.001.

Since cardiomyopathy develops at a later age in *mdx* mice, we did not measure heart function at 50 days. We did not see significant differences in the left ventricular ejection fraction and shortening fraction measures at 100 days of treatment, but these measures were significantly reduced in the GC-treated group at 180 days (p<0.01and p<0.001, respectively; Figure 3A, B). The rate of decline in cardiac function in the GC-treated group was much more rapid than that in the untreated group between 100 and 180 days of treatment. Evaluation of normalized left ventricular volumes indicated that at 100 days, GC-treated mice had significantly (p = 0.016) larger normalized volumes (2.46±0.17) than did untreated (1.89±0.16) mice, indicative of a dilated cardiomyopathy. There was a significant increase in cardiac fibrosis in the GC-treated group at both 100 (p<0.05) and 180 (p<0.01) days (Figure 3C).

### Effect on in Vitro Force Contraction

Since behavioral measures are subject to the animal’s volitional efforts, we have measured in vitro force contractions on isolated EDL muscle. In comparison to the EDL muscle from the untreated mice, the maximum force (mN) in the EDL muscle from the GC-treated mice was significantly higher at 100 days (p<0.3), but this effect was eventually lost at 180 days (p = 0.18). GC treatment reduced the EDL muscle mass. The specific force (KN/m^2^) did not differ between the two groups at either 100 or 180 days (Table 3).

**Table 3:** Muscle force at 100 and 180 days in untreated and GC-treated mice.

Time(days)	Measurement	GC treated group		Untreated group		p-value fortreatment group
		N	Mean ± SEM	N	Mean ± SEM	
∼100	Maximum force (mN)	20	319±12	20	260±12	0.027
	Specific force (kN/m^2^)	20	158±5	20	157±5	0.925
	EDL muscle mass (mg)	20	13.1±0.3	20	14.7±0.3	0.002
∼180	Maximum force (mN)	6	374±22	6	418±22	0.182
	Specific force (kN/m^2^)	6	192±12	6	200±12	0.640
	EDL muscle mass (mg)	6	12.8±0.3	6	13.6±0.3	0.072

### Effect on Skeletal Muscle Histology

**Figure 4:**
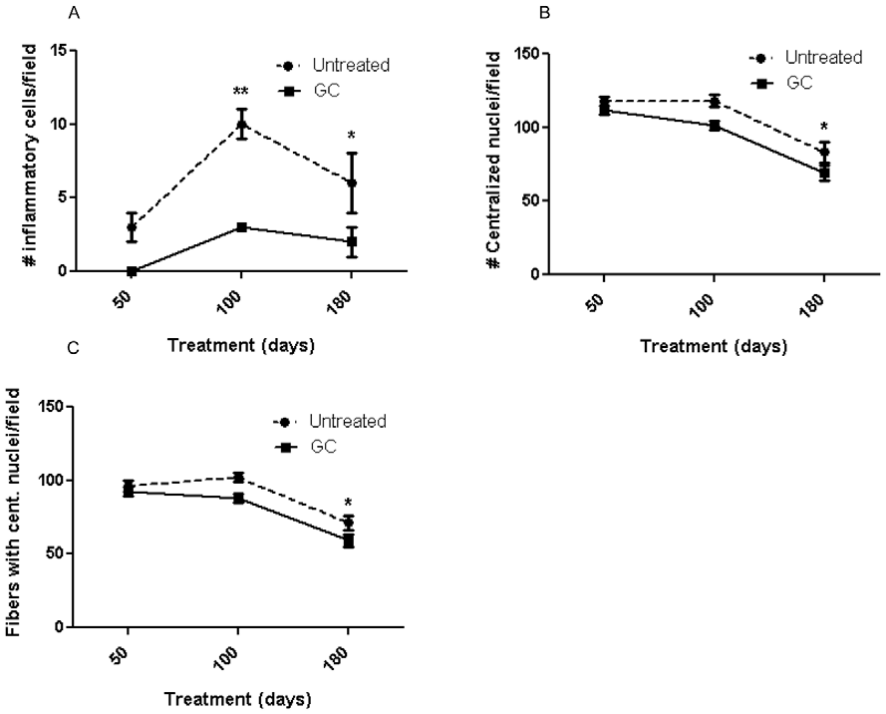
Hematoxylin and eosin (H & E) evaluation of skeletal muscle. H&E staining was carried out to assess the effect of treatment on inflammation (**A**), central nucleation (**B**), and fibers with central nucleation (**C**). Inflammatory cells were quantified in five non-overlapping fields in gastrocnemius muscle. *mdx* mice received no treatment (N = 4, 15, and 15) or GC treatment (N = 4, 18, and 16) for 50, 100, or 180 days, respectively. Error bars indicate mean+/- SEM. * p  =  <0.05 and ** p  =  <0.01.

Examination of H&E-stained sections of gastrocnemius muscle from the GC-treated mice showed a significant decrease in the number of inflammatory cells at 100 (p<0.01) and 180 (p<0.05) days and in the number of total central nuclei (p<0.05) and number of fibers with central nuclei (indicating cells undergoing regeneration) at 180 days (p<0.05), when compared to the untreated mice (Figure 4A-C).

## Discussion

In this study, we have taken a comprehensive approach to evaluating the effects of chronic administration of GC on the disease phenotype in *md*x mice. Our data indicate that continuous administration of GCs significantly improves the disease phenotype (normalized behavioral parameters) early in the disease but that these beneficial effects are eventually lost with continued treatment. In addition, our data indicate that prolonged GC administration significantly decreases heart function and increases heart fibrosis, indicating that prolonged GC treatment is detrimental to dystrophic heart and skeletal muscle and further suggesting that these drugs may not be appropriate positive therapeutic controls for long-term preclinical drug testing in this mouse model.

Since body weight is a simple measure of the overall drug effect on the mouse phenotype, we measured body weight and found a significant decrease in the body weight of *mdx* mice. GCs are known to induce catabolic effects in skeletal muscle by activating the ubiquitin-proteosome pathway. It has previously been demonstrated that muscle-specific E3 ligases (Atrogin-1/MAFbx and Murf1) are up-regulated in response to GC administration in skeletal muscle [9].

A decrease in body weight and reduction in weight gain with GC treatment are well-documented in the literature: For example, Keeling et al. performed an 84-week trial of twice-weekly oral prednisolone and followed the survival of the treated *mdx* mice through 104 weeks of age. They found that the treated mice survived longer and had a slower decline in grip strength per gram of body weight than did the untreated *mdx* mice [10]. Similar observations were also made by Lefaucheur et al. [11]. However, Keeling’s study did not show any significant improvement in grip strength between 3 and 10 weeks under non-exercise conditions [10]. Granchilli et al. found a 24% increase in strength (normalized to body weight) in *mdx* mice that were treated with 1mg/kg prednisone for 6 weeks and subjected to forced treadmill exercise (20cm/sec) for 30 min twice a week [12]. These authors found that higher doses of prednisone (5, 10, and 20mg/kg) were detrimental to strength. This result is consistent with our finding that 50-day treatment with 1mg/kg of GCs significantly improved normalized forelimb grip strength and that the rate of decline was slower than that in untreated mice, suggesting the beneficial effects of drug treatment persist up to 100 days but become non-significant by 180 days. Granchelli and colleagues reported greater strength, decreased fatigue, and increased muscle fiber diameters in treated mice, suggesting a protective effect of GCs [12]. In 2007, Golumbek et al. similarly described an improved performance per body weight and in running speed in *mdx* mice receiving GC treatment, but they also reported an incremental increase in the frequency of calcifications [5]. Another study by Yang et al. examined the effect on diaphragm function of a 6-week (2.25 mg/kg) treatment with methylprednisolone [13]. They found a slight improvement in the contractile properties of the treated mice; however, they did not look at skeletal muscle function.

The present study has shown that prolonged steroid use leads to decreased cardiac systolic function and increased cardiac fibrosis in *mdx* mice. These results are in agreement with those of Bauer et al., who found that 1.5 mg/kg/day prednisolone delivered via drinking water over 8 weeks in 4-month-old *mdx* mice resulted in increased left ventricular dilatation, decreased diastolic function, and increased cardiac fibrosis [14]. Previous work from our laboratory has also demonstrated significantly decreased cardiac function and increased cardiac fibrosis in a subcutaneous, continuous prednisone delivery protocol at a dose of 1 mg/kg/day [8]. Skrabek and Anderson treated 2- to 3-month-old *mdx* mice daily with intraperitoneal prednisone (1 mg/kg) or deflazacort (0.65 and 1.2 mg/kg) and found significantly decreased fibrosis only in the high-dose deflazacort-treated *mdx* mice. The low-dose deflazacort and prednisone (equipotent with the high-dose deflazacort) did not show any significant differences from untreated mice. Thus, prednisone treatment did not increase cardiac fibrosis in this study, in part because of the short duration of the study [15]. However, an earlier study by Marques et al. found a decrease in cardiac fibrosis in 6-month-old *mdx* mice treated with 1.2 mg/kg of deflazacort in the drinking water for 15 months [16]. These results are in direct contrast to our current study and the others previously mentioned. The differences could be due to the difference in drug (deflazacort vs prednisone/prednisolone), sex, age, and duration of drug administration. It also appears that long-term administration of deflazacort has negative consequences on heart function in the delta-sarcoglycan-deficient cardiomyopathic hamster, suggesting that careful studies are needed to confirm and validate the previously reported beneficial effects of these drugs in *mdx* mice [17]. It is also worth noting that most animal studies use more-continuous delivery methods (drinking water, chow, subcutaneous pellet) than do clinical studies, and this continuous administration may be more deleterious than the single-dose therapies used clinically in human patients. Recent evidence has shown that pulsed steroids can resynchronize cell cycling and cytokine signaling, leading to decreased inflammation after injury in an asthma cell culture model [18]. Perhaps an optimal GC dosing schedule could help minimize inflammation, whereas other GC dosing schedule would increase it. Further studies in animal models should more closely replicate clinical dosing schedules to allow us to better assess any deleterious effects.

We also assessed the overall behavioral activity of the mice using an open-field Digiscan apparatus and found that the animals adapted to the Digiscan apparatus over a period of time and generally performed better with time than they did at the initial time point. A comparison of the treatment groups clearly indicated that the GC-treated group generally showed higher behavioral activity measures (grip strength, Rotarod performance, open-field activity) when the GC was normalized to body weight in early stages of the treatment. The beneficial effects are clearly related to the loss of body weight that occurs upon GC treatment. Many of beneficial effects are abolished by 180 days, indicating that even the normalized beneficial effects are transient. It is likely that the side effects of GC overtake the beneficial effects after prolonged GC treatment in dystrophin-deficient mice.

Taken together, these data point to beneficial effects of GCs early in the period of administration but also to deleterious effects associated with chronic administration. It is generally accepted that therapy with corticosteroids improves the muscle strength of patients and benefits their ambulation, scoliosis, and respiratory function. The treatment has only engendered problems with weight gain as the principal side effect. Results similar to ours have been reported by Granchelli et al, who found that a dose of 1 mg/kg/d of prednisone improved strength in *mdx* mice by 24%, whereas higher doses ranging from 5 to 20 mg/kg were deleterious [12].

Our findings in mice point out clear differences from the human experience with GCs in DMD patients. Based on the recommendations of an international workshop in 2004, daily steroid therapy has become the “gold standard” treatment in DMD patients [19]. These recommendations had been based on many studies that showed the benefits of steroids on skeletal and respiratory muscle function [20], [21], [22], [23]. Studies then began to focus on the cardiac effects. Silversides et al. showed that only 5% of patients treated with deflazacort for 3 or more years had a significantly decreased ejection fraction, as compared to 58% of untreated patients [24]. They also found a correlation between preservation of cardiac function and improvement in pulmonary and skeletal muscle function. Markham et al. found that steroid-naïve subjects 10 years old or younger were 4.4 times more likely to have decreased cardiac function, and those older than 10 years were 15.2 times more likely to have decreased function [25]. Of particular interest is the fact that patients who had received steroids but were no longer taking them showed normal cardiac function and no differences from patients who continued to receive steroids. Biggar et al. reported that 59% of their untreated DMD patients developed decreased cardiac function by 18 years of age, as compared to 10% of steroid-treated patients [23]. Houde et al. found that deflazacort treatment preserved cardiac function in DMD patients over an 8-year follow-up study [26]. Treated patients had improved systolic function and a decreased incidence of dilated cardiomyopathy (32% vs. 58%) when compared to younger untreated patients. Markham et al. reported that 93% of steroid-treated DMD children maintained normal cardiac function, as compared to only 53% of untreated children [25]. These studies support the idea that DMD patients treated with steroids prior to the onset of cardiac dysfunction show a slower progression of heart disease. The study by Markham et al. also questioned whether there is an early therapeutic window for obtaining the beneficial effects of steroid therapy in cardiac muscle [25]. However, questions still remain regarding the best type of steroid to use and the optimal age of therapy initiation, dosing schedule, and duration of therapy. Further studies addressing these questions and utilizing improved cardiac outcome measures are clearly needed.

The significant increase in collagen deposition that we saw in the GC-treated group suggests that long-term treatment with GCs is detrimental to heart function, not only in the *mdx* model but in other models of muscular dystrophy in which heart damage is evident in the early stages [27]. The amount of collagen observed in the heart was significantly higher in the GC-treated group than in the control group at either 100 or 180 days, and similar results were found in the EDL at 100 days (data not shown). However, Hartel in 2001 reported an advantage of prednisone in reducing TGF-β and hydroxyproline levels in the diaphragm, suggesting a potential involvement in the genesis of fibrosis [28]. In contrast, no significant differences were found in CK. In 1991, Weller reported that methylprednisolone failed to significantly influence the time course and prevalence of necrosis and regeneration, although it produced lower CK values [29].

There is no doubt that steroids are beneficial for young patients with DMD and are the standard of care for this disease. Interestingly, *mdx* mice do not seem to benefit substantially from steroid treatment despite a resulting decrease in skeletal muscle inflammation. We want to point out that treatment effects in mouse models may be different from the effects one would observe in patients. The steroid dose we used in our study was slightly higher than that normally used for children with DMD, and the steroids were not administered in a single oral dose but rather in the chow or in slow-release pellets, a difference that might affect their pharmacodynamic effect and consequently the side effects.

In conclusion, our data strongly demonstrates that chronic continuous daily administration of GCs to *mdx* mice significantly improves strength in the initial stages of the disease, but it also causes deterioration in muscle and heart function, increases fibrosis, and contributes to the deterioration of overall activity in this model. This functional improvement in grip strength is not correlated with histological decrease in inflammation: GC-treated mice show a decrease in inflammation both at 100 and 180 days, without marked improvement in functional measures, suggesting that decreased inflammation alone is unlikely to improve the function of dystrophin-deficient muscle. Thus, there is an urgent need for the development of compounds that provide the anti-inflammatory potency of standard GCs but with markedly reduced side effects.
